# GhYGL1d, a pentatricopeptide repeat protein, is required for chloroplast development in cotton

**DOI:** 10.1186/s12870-019-1945-1

**Published:** 2019-08-13

**Authors:** Peng He, Shuyin Wu, Yanli Jiang, Lihua Zhang, Meiju Tang, Guanghui Xiao, Jianing Yu

**Affiliations:** 1 College of Life Sciences, Shaanxi Normal University, Xi’an, 710119 China; 2 Shanxi Academy of Agricultural Sciences, Cotton Research Institute, Yucheng, 044000 China; 3 Key Laboratory of the Ministry of Education for Medicinal Plant Resources and Natural Pharmaceutical Chemistry, National Engineering Laboratory for Resource Development of Endangered Crude Drugs in the Northwest of China, College of Life Sciences, Shaanxi Normal University, Xi’an, 710119 China

**Keywords:** Chloroplast, PPR, Leaf variegation, Cotton

## Abstract

**Background:**

The pentatricopeptide repeat (PPR) gene family, which contains multiple 35-amino acid repeats, constitutes one of the largest gene families in plants. PPR proteins function in organelles to target specific transcripts and are involved in plant development and growth. However, the function of PPR proteins in cotton is still unknown.

**Results:**

In this study, we characterized a PPR gene YELLOW-GREEN LEAF (*GhYGL1d*) that is required for cotton plastid development. The *GhYGL1d* gene has a DYW domain in C-terminal and is highly express in leaves, localized to the chloroplast fractions. GhYGL1d share high amino acid-sequence homology with AtECB2*.* In *atecb2* mutant, overexpression of *GhYGL1d* rescued the seedling lethal phenotype and restored the editing of *accD* and *ndhF* transcripts. Silencing of *GhYGL1d* led to the reduction of chlorophyll and phenotypically yellow-green leaves in cotton. Compared with wild type, *GhYGL1d*-silenced cotton showed significant deformations of thylakoid structures. Furthermore, the transcription levels of plastid-encoded polymerase (PEP) and nuclear-encoded polymerase (NEP) dependent genes were decreased in *GhYGL1d*-silenced cotton.

**Conclusions:**

Our data indicate that *GhYGL1d* not only contributes to the editing of *accD* and *ndhF* genes, but also affects the expression of NEP- and PEP-dependent genes to regulate the development of thylakoids, and therefore regulates leaf variegation in cotton.

**Electronic supplementary material:**

The online version of this article (10.1186/s12870-019-1945-1) contains supplementary material, which is available to authorized users.

## Background

Life on earth depends on the process of photosynthesis, which converts light energy into chemical energy, in chloroplasts to provide us with food and oxygen [1]. Chloroplasts are specialized organelles in plants and an abundance of chloroplasts are found in the leaf, which is the primary location of photosynthesis and sugar manufacturing. The development of a leaf directly affects photosynthetic efficiency of an individual plant, which further determines the yield of plants [2]. Dozens of studies have demonstrated that coordinated expression and regulation between both nuclear and chloroplast genes is very important for biogenesis of chlorophyll and the development of chloroplasts [3]. Numerous nuclear-encoded genes were found to be involved in RNA processing events such as editing, splicing and degradation of chloroplast genes [4–8]. As nuclear factors, dozens of PPR proteins have been proved to be involved in the expression of chloroplast genes [9, 10].

The PPR protein family was one of the largest protein families in plants, which contained several repeating motifs consisting of 35 amino acids [11, 12]. In land plants, the PPR family has more than 400 protein members [13]. Proteins in the PPR family in plants are classified into two major subfamilies according to their different motifs [14]. The P subfamily of proteins contain only the P motif, while the PLS subfamily consists of degenerated P, L, and S motifs, where the S motif has 31 amino acids and L motif has 35 or 36 amino acids [15]. PLS subfamily proteins are plant-specific and always possess C-terminal domains named E, E+ and DYW, which reportedly are involved in organelle RNA editing in plants [16]. Most PPR proteins are anchored into either the mitochondria or the chloroplast. There are 466 PPR proteins found in the *Arabidopsis thaliana* genome. Among them, 88 members belong to PPRs with the DYW domain [17].

Pentatricopeptide repeat proteins are involved in many post-transcriptional processes in chloroplasts and mitochondria, including RNA cleavage [18, 19], alternative splicing [20–22], transcriptional regulation [23, 24], RNA editing [25, 26], mRNA stabilization [27, 28], and translation [29, 30]. The PPR protein EMP9 with 16 PPR motifs is required for the editing of mitochondrial *ccmB-43* and *mrps4-335*, which affect seed development in maize [31]. The MEF13 protein, which consists of 21 PPR motifs, is required for RNA editing at eight sites in mitochondrial mRNAs in *Arabidopsis* [32]. CHLORORESPIRATORY REDUCTION 4 (CRR4), the first RNA-editing factors with 11 PPR repeats identified in chloroplasts, is responsible for RNA editing of the initiation codon of *ndhD* [33]. Growing slowly 1 (GRS1), a PLS-type PPR protein, is involved in RNA editing at four specific sites and affects plant development [34]. *Arabidopsis* PDM1/SEL1 encode a PLS-type PPR protein, the *pdm1* loss-of-function mutant exhibits pigment-deficient phenotype with a defect in RNA splicing of *trnK* and *ndhA* [35]. PDM2, a plastid-localized PPR, plays an important role in the accumulation of plastid-encoded transcripts and plastid RNA editing [36]. PDM3 is a chloroplast protein with 12 PPR repeats domains and responsible for the RNA splicing of *trnA*, *ndhB*, and *clpP1* transcript [37]. In rice, a plastid-localized PPR protein, OsPPR6, reportedly mediates both RNA editing and splicing [38].

The DYW domain is named for the frequent presence of an Asp-Tyr-Trp tripeptide at the C terminal, and it contributes to the discrimination of target and non-target editing sites [39]. The *AtECB2* gene, encoding a PPR protein with a C-terminal DYW domain, is required for editing of *accD* genes and chloroplast biogenesis [40]. In *Arabidopsis*, DYW proteins CRR22 and CRR28 play important roles in RNA editing and RNA cleavage [13]. The *YS1* gene encoding a DYW protein is essential for editing of *rpoB* transcripts and the development of leaves [41]. The DYW domain of the OTP85 protein catalyzes site-specific cleavage and editing of target RNA [42]. In moss, *PPR_71* gene, encoding a PPR protein with a DYW domain, is required for RNA editing of the *ccmFc* transcript [43]. The *PPR_43* gene, which encodes a mitochondrial-localized PPR protein with a C-terminal DYW domain, is responsible for the splicing of *cox1* pre-mRNA at the second intron [44]. In rice, OGR1, a PPR-DYW protein, is essential for RNA editing in mitochondria and is normal growth and development [45].

Cotton is the most important textile fiber and also a significant oilseed crop. The first report about PPR proteins in cotton was the identification of five PPR proteins in upland cotton [46]. Furthermore, eight PPR family genes were cloned and their expression patterns investigated [47]. Recently, a genome-wide identification of PPR genes with DYW domains was described [48]. However, the biological function of PPR proteins in cotton is largely unknown.

In this study, using computational prediction followed by verification with a virus-induced gene silencing (VIGS) experiment, we identified a PPR-DYW family gene, *GhYGL1d*, which was essential for cotton leaf development. Overexpression of *GhYGL1d* in *Arabidopsis* mutant, *atecb2,* resulted in a similar phenotype as the wild type plants. Reduction of *GhYGL1d* transcripts decreased the content of chlorophyll, led to the disruption of thylakoid structure and produced albino leaves in cotton. In addition, we also found that silencing the *GhYGL1d* gene caused dramatic reductions in transcription levels of PEP-dependent genes. Our findings will not only characterize mechanisms of cotton leaf variegation regulated by *GhYGL1d*, but also provide the strategy to study the function of other PPR-DYW genes in the future.

## Results

### Screening and identification of the GhYGL1d gene

**Fig. 1:**
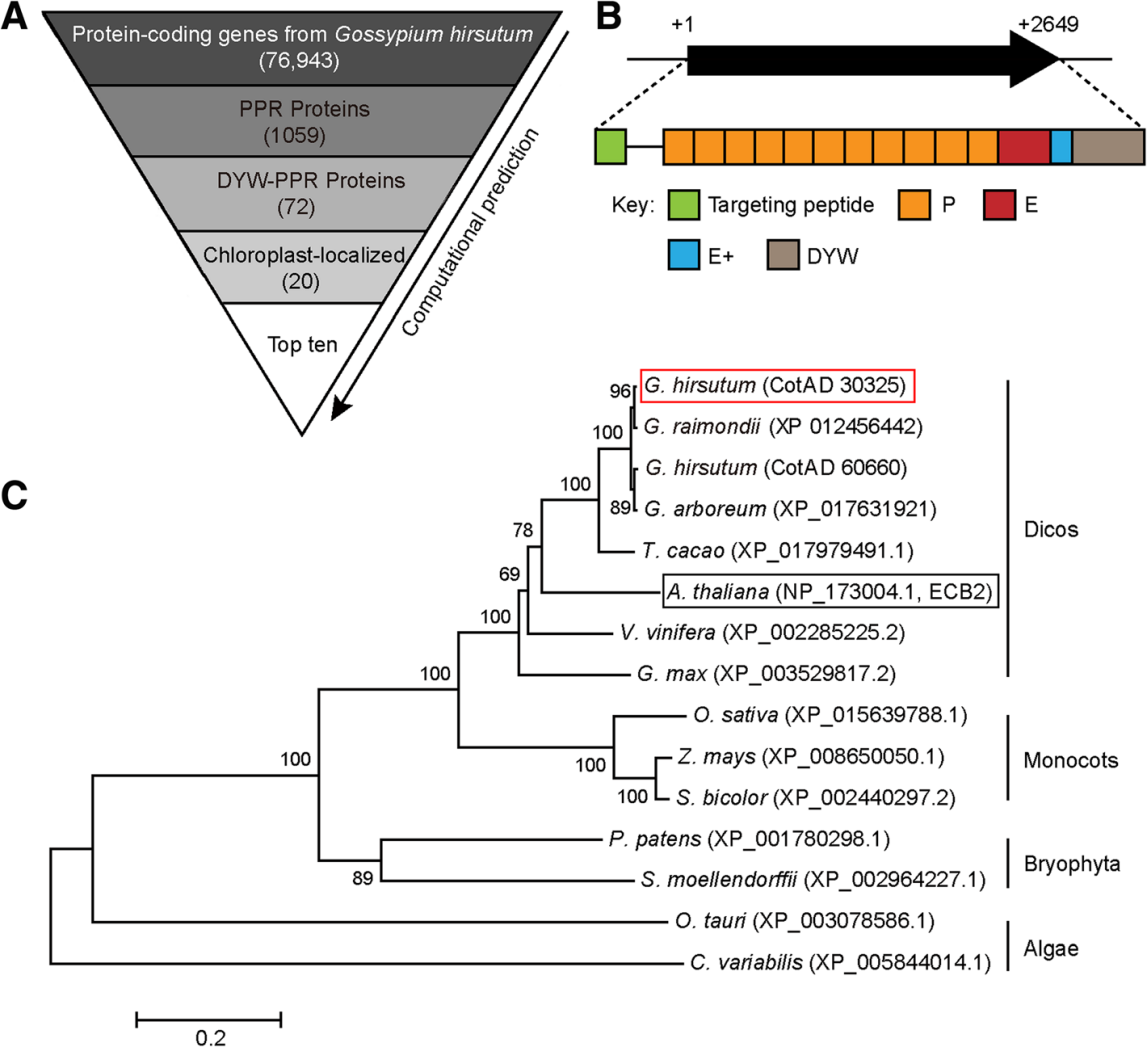
Phylogenetic and sequence analysis of GhYGL1d and its expression pattern. **a** Computational prediction for identification of leaf-related RNA editing factors in cotton. **b** Schematic structures of GhYGL1d proteins. Predicted targeting peptide, P, E, E+ and DYW domains are labeled on the protein sequence. The targeting peptide was predicted using the TargetP software (www.cbs.dtu.dk/services/TargetP/). The PPR motifs and domains were predicted by TPRpred software (https://toolkit.tuebingen. mpg.de/#/tools/tprpred). **c** Phylogenetic tree analysis of GhYGL1d proteins in plants were performed using the MEGA program (www.megasoftware.net). The phylogenetic tree was generated by MEGA5.0. **d** YGL1 protein in *Gossypium raimondii* (XP_012456442), *Gossypium arboreum* (XP_017631921), *Theobroma cacao* (XP_017979491.1), *Arabidopsis thaliana* (NP_173004.1), *Vitis vinifera* (XP_002285225.2), *Glycine max* (XP_003529817.2), *Oryza sativa* (XP_015639788.1), *Zea mays* (XP_008650050.1), *Sorghum bicolor* (XP_002440297.2), *Physcomitrella patens* (XP_001780298.1), *Selaginella moellendorffii* (XP_002964227.1), *Ostreococcus tauri* (XP_003078586.1), and *Chlorella variabilis* (XP_005844014.1) were selected to generate a bootstrap neighbor–joining phylogenetic unrooted tree. The numbers at each node represent the bootstrap values (%) calculated from 1,000 trials. The length of branches indicates the extent of divergence according to the bar scale (relative units) at the bottom.

PPR-DYW proteins play important roles in many biological processes, especially in RNA editing events [49]. To identify all of the PPR-DYW proteins in G. hirsutum, we screened for all PPR proteins in the cotton genome database (https://www.cottongen.org) and a total of 1,059 PPR proteins were identified (Fig. 1a). Among them, 72 PPR-DYW proteins with 20 members localized in chloroplast were found (Additional file 1 Table S1). We selected ten PPR-DYW proteins with cTP values more than 0.6 (predicted by ChloroP or TargetP program) to investigate their biological functions using the VIGS experiment. We amplified nine gene fragments with specific primers (Additional file 1 Table S2) to silence their expression. In contrast to the silenced cotton with an empty vector, the *CotA_30325*-silenced plant displayed variegated leaf phenotypes (Additional file 1 Figure S1). An analysis of the CotA_30325 protein in the Pfam database showed that it contained a tandem repeat of 11 PPR motifs and belonged to a member of the DYW subfamily (Fig. 1b). A BLAST search of the NCBI database showed that *CotAD_30325* is a double-copy gene in the cotton genome which shared 98.98% similarity with *CotAD_60660* in their cDNA sequence over the 2649 bp length. To better present the evolutionary relationship among CotAD_30325 proteins, we constructed a phylogenetic tree and found that the CotA_30325 protein shared high sequence homology with AtECB2 in *Arabidopsis* (Fig. 1c). According to the phylogenetic relationships, the *CotAD_30325* gene was from the D-subgenome named as *GhYGL1d*. We also performed an amino acid sequence alignment of GhYGL1d and AtECB2 and the result showed that GhYGL1d shared 11 conserved PPR motifs with AtECB2 (Additional file 1 Figure S2).

### Expression pattern and subcellular localization of GhYGL1d

**Fig. 2:**
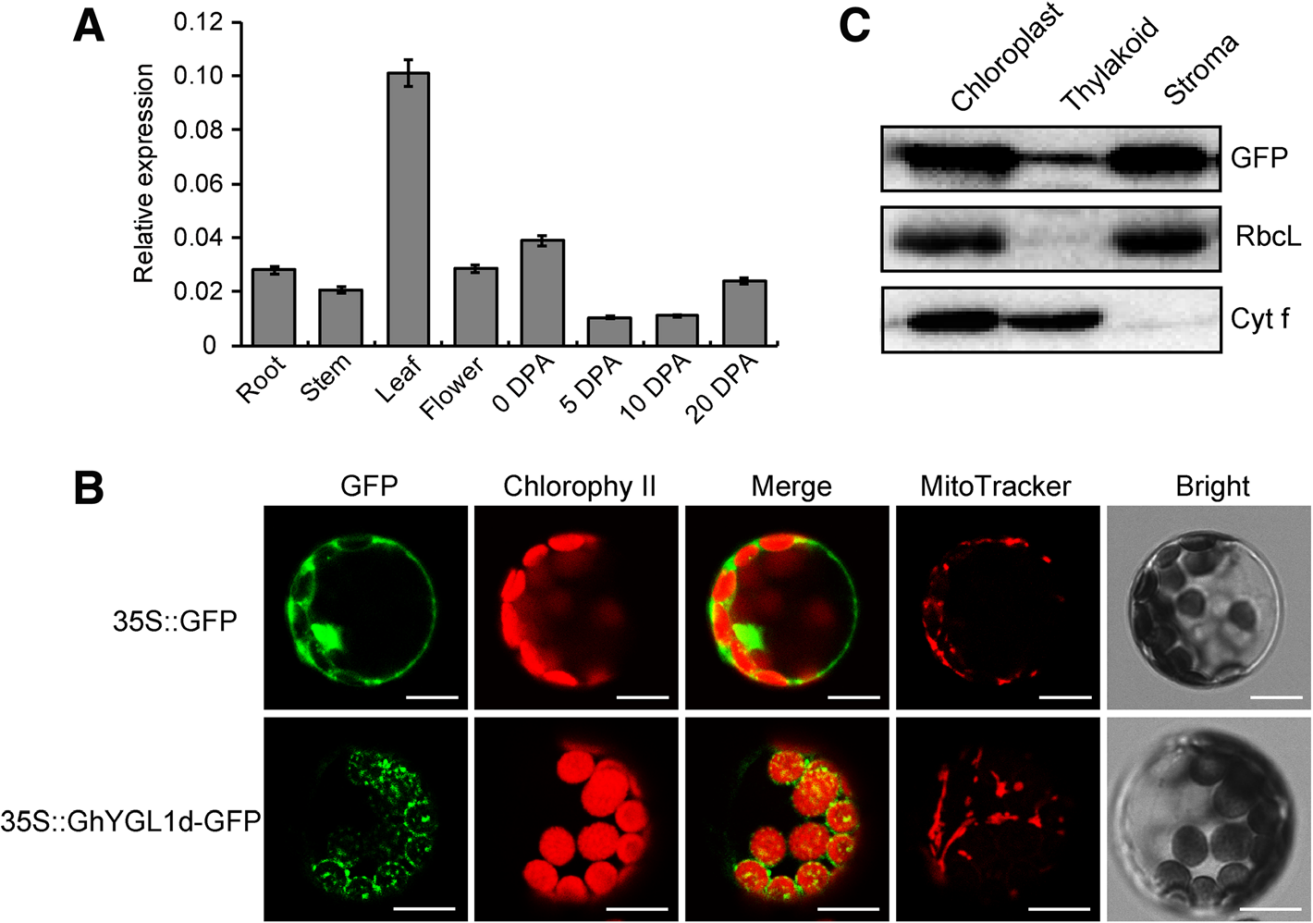
Expression and subcellular localization of GhYGL1d. **a** qRT-PCR analysis of *GhYGL1d* expression in different tissues. Total RNA was isolated from roots, stems, leaves, flowers and fibers. The *GhUBQ7* gene was used as a reference gene for qRT-PCR. DPA, day post-anthesis. The values shown are means ± SE of three replicates. **b** GhYGL1d localizes to the chloroplast. Protoplasts from wild-type *Arabidopsis* were transiently transformed with a control GFP vector (designated 35S::GFP) or with a GhYGL1d-GFP vector. Fluorescence was observed by confocal microscopy of single protoplasts; green fluorescence = GFP; red = chlorophyll autofluorescence. Bars = 10 μm. **c** GhYGL1d localizes to the stroma and thylakoid fractions of chloroplasts. Total proteins extracted from the 35S::GhYGL1d-GFP transgenic line were used to confirm the specificity of the anti-GFP antibody. Intact chloroplasts were isolated from 35S::GhYGL1d-GFP transgenic seedlings and separated into the thylakoid and stroma fractions. Chloroplasts protein Cyt f and RbcL were used as a marker of thylakoid membrane and stroma fractions, respectively. The GFP antibody was used to detect the GhYGL1d-GFP fusion protein.

In order to understand the potential physiological functions of *GhYGL1d* gene in cotton, the expression patterns of the GhYGL1d gene were investigated in various tissues. Results show that GhYGL1d was highly expressed in leaf tissues (Fig. 2a), suggesting that GhYGL1d may play an important role in the leaf, which is consistent with the result of the leaf phenotype in the GhYGL1d-silenced plant.

GhYGL1d is predicted to be targeted to the chloroplast by Target P (http://www.cbs.dtu. dk/services/TargetP/). To confirm this prediction, a construct with the full-length *coding sequence* of GhYGL1d fused to green fluorescent protein (GFP) driven by the CaMV 35S promoter was transiently expressed in Arabidopsis protoplasts. Using confocal laser scanning microscopy, we *observed that* GhYGL1d-*GFP fusion protein signals* highly overlapped with the red auto-fluorescent signals of chlorophyll *in the protoplasts (*Fig. 2b). To further elucidate the *precise* location of GhYGL1d in chloroplasts, we generated transgenic plants expressing the full-length sequence of *GhYGL1d* fused with GFP. We extracted chloroplasts from transgenic plants and separated the chloroplast into thylakoid membrane and stroma fractions. Strong immunoblotting signals were observed in the stroma fraction, which suggests that GhYGL1d highly accumulated in the stroma fraction of plastids (Fig. 2c).

### **Expressing GhYGL1d in an atecb2 mutant restores the defect**

**Fig. 3:**
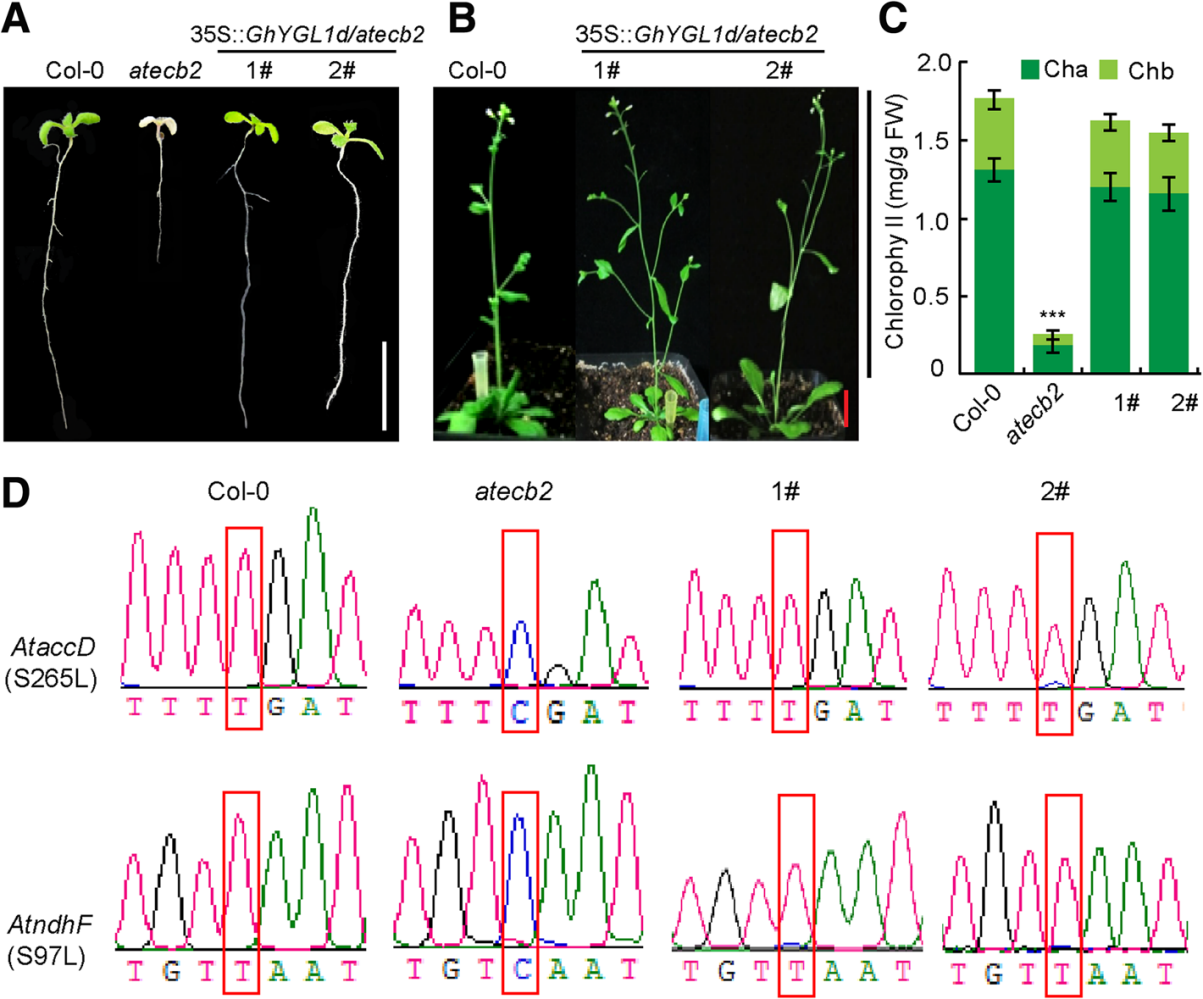
*GhYGL1d* partially restored the function of *AtECB2* in *Arabidopsis*. (A, B) Phenotypes of a wild-type (Col-0) plant, an *atecb2* mutant and GhYGL1d complemented plants at two (**a**), and six (**b**) weeks after sowing. Scale bars are 2 cm. (C) Chlorophyll contents in a wild-type (Col-0) plant, an *atecb2* mutant and GhYGL1d complemented plants. FW, fresh weight. Chl a, chlorophyll *a*. Chl b, chlorophyll *b.* Error bars indicate SD for three biological replicates. Asterisks indicate significant differences (*P* < 0.001) from Col-0 plants. (D) RNA editing of *accD*, *ndhF* transcripts in wild-type (Col-0) plants, *atecb2* mutants and GhYGL1d complemented plants. 1# and 2# indicate GhYGL1d complemented lines.

In A. thaliana, the atecb2 mutant displayed albino cotyledons and unorganized chloroplast structure. Moreover, the RNA editing at accD-794 and ndhF-C290 sites was abolished. *T*o test the function of GhYGL1d, we expressed GhYGL1d, driven by the constitutive 35S promoter, in the atecb2 mutant. After confirming by the PCR method, we successfully obtained the transgenic plant. As shown in Fig. 3, complement 35S::GhYGL1d mutant plants restored chlorophyll production, avoiding the production of albino cotyledons (Fig. 3a), and also seedlings were rescued from lethality (Fig. 3b). At the same time, the atecb2 mutant expressing GhYGL1d produced almost identical amounts of chlorophyll as compared with the wild-type plants (Fig. 3c). In the atecb2 mutant, the editing of accD and ndhF transcripts was deficient, which resulted in an alteration of the amino acid serine to leucine in AccD and NdhF proteins, respectively [50]. Given that GhYGL1d could rescue the phenotype of the atecb2 mutant, it is likely that GhYGL1d restored the editing of accD and ndhF transcripts. To test this, we examined the editing sites of accD and ndhF transcripts in wild-type, atecb2 mutant and GhYGL1d complemented plants. Indeed, the editing sites of accD and ndhF in the transgenic plants were identical to that in wild-type plants (Fig. 3d). These results indicate that GhYGL1d can restore the function of AtECB2 in Arabidopsis.

### **GhYGL1d is required for chloroplast development**

**Fig. 4:**
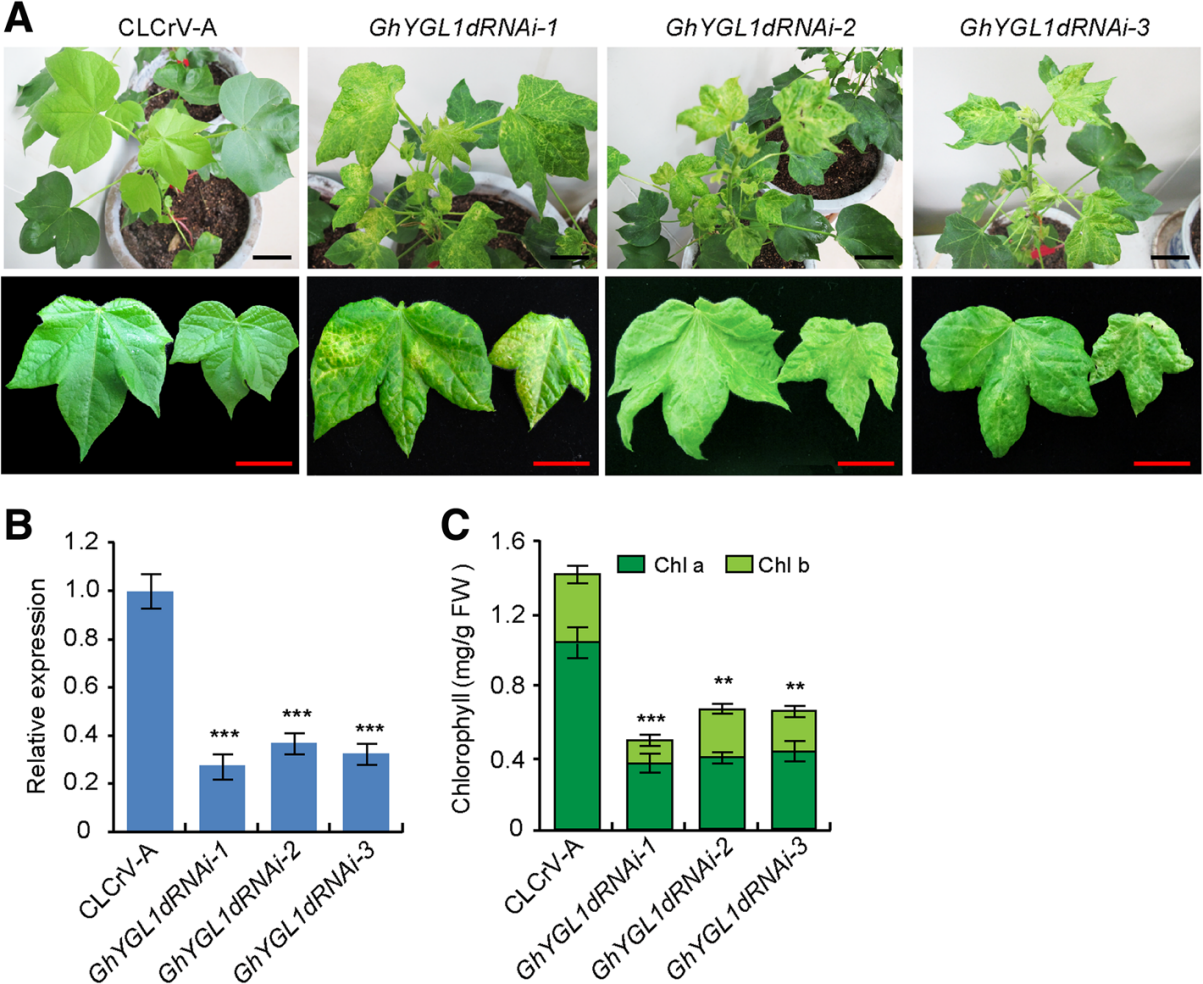
Silencing of *GhYGL1d* showed variegated leaves. **a** Cotton plants infiltrated with CLCrVA:*GhYGL1d* (*GhYGL1d-RNAi*) showed variegated leaves. The photographs were taken at approximately five weeks after infiltration. Wildtype plants transformed by an empty CLCrVA vector was used as the control. Scale bars are 2 cm. **b** qRT-PCR analysis of GhYGL1d transcripts in RNAi plants. The *GhUBQ7* gene was used as a reference. The values shown are means ± SE of three biological replicates. Significant differences between RNAi plants and CLCrVA control plants were calculated using Student’s t-test: ***, *P* < 0.001; (C) Chlorophyll contents in gene-silenced plants. FW, fresh weight. Chl a, chlorophyll *a*. Chl b, chlorophyll *b.* Values are means ± SD of three replicates. Student’s t-test: ***, *P* < 0.001;

**Fig. 5:**
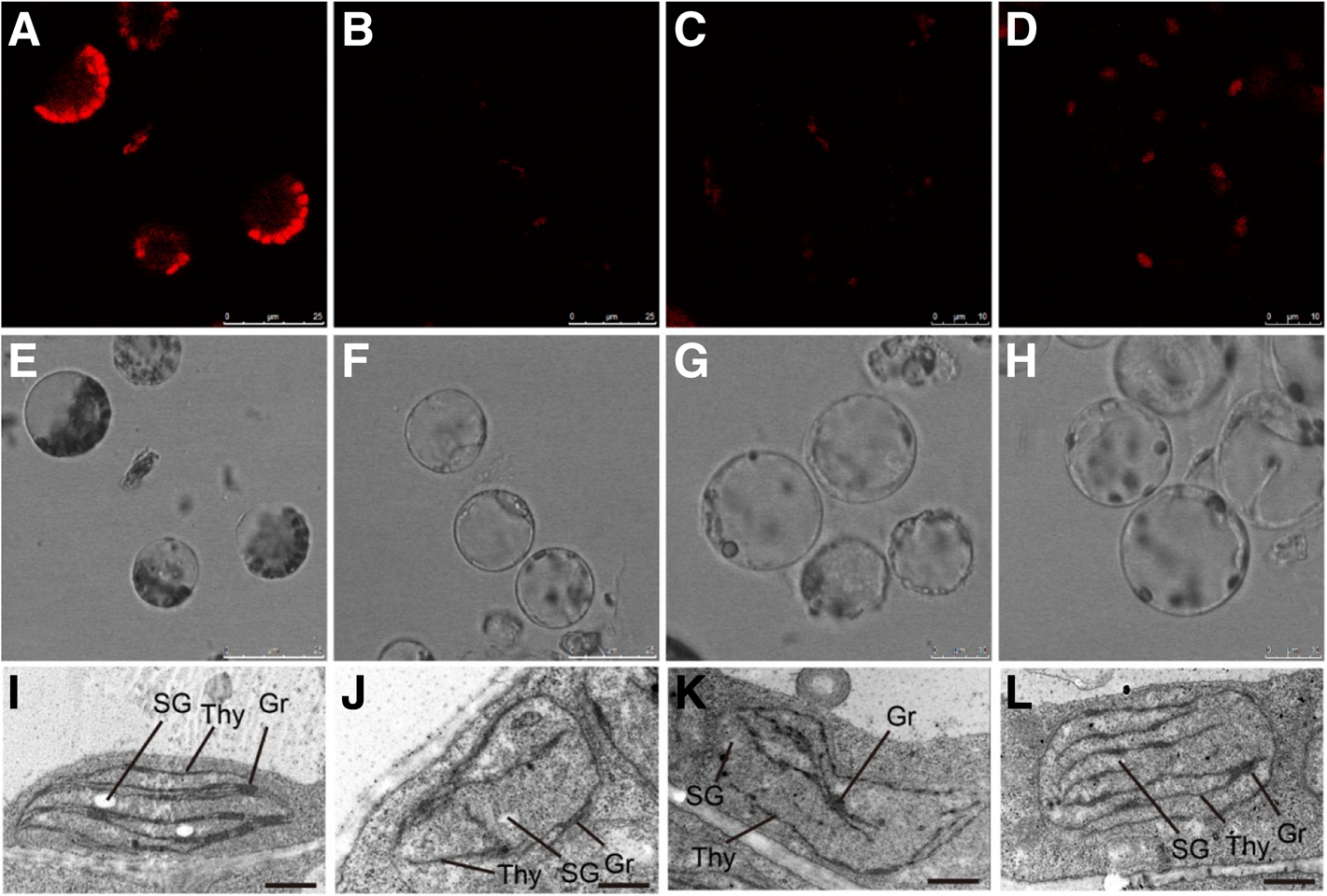
Loss of GhYGL1d expression affects chloroplast development. **a**-**h** Chlorophyll fluorescence and morphology of the protoplasts isolated from the CLCrVA (**a**, **e**), GhYGL1d-RNAi-1(**b**, **f**), GhYGL1d-RNAi-2 (**c**, **g**) and GhYGL1d-RNAi-3 (**d**, **h**) plant leaves. (**i**-**l**) Transmission electron micrographs of plastid ultrastructures in the CLCrVA (I), GhYGL1d-RNAi-1(J), GhYGL1d-RNAi-2 (K) and GhYGL1d-RNAi-3 (L) plants. Plastids were from leaves of six-week-old plants. Three biological replicates were performed, and similar results were obtained. SG, starch grain. Thy, thylakoid. Gr, granum. Bars = 1 μm.

To further elucidate the possible function of GhYGL1d in cotton chloroplast development, A. tumefaciens containing the *CLCrVA*-GhYGL1d construct was infiltrated into wild-type cotton cotyledons. Four weeks later, the cotton plants infiltrated with *CLCrVA*-GhYGL1d showed variegated leaves unlike the normal leaf phenotype of plants infiltrated with the empty vector (Fig. 4a). We detected the transcript level of GhYGL1d and the RT-qPCR experiments showed that the transcripts of GhYGL1d significantly decreased in CLCrVA-GhYGL1d RNAi lines (Fig. 4b). In addition, the contents of both chlorophyll a and b in CLCrVA-GhYGL1d RNAi plants were reduced in contrast to that in control plants (Fig. 4c). And the chlorophyll fluorescence of protoplasts isolated from the CLCrVA-GhYGL1d RNAi plants also showed a decreased intensity (Fig. 5a-h). We also compared the ultrastructure of chloroplasts by TEM. The chloroplasts of control plants were crescent-shaped and contained obvious starch grains and well-formed thylakoid structures, while in GhYGL1d RNAi plants, the chloroplasts *showed an unorganized chloroplast structure* (Fig. 5i-l)***.*** Taken together, our data suggest that GhYGL1d is required for leaf chloroplast development.

### **GhYGL1d effects on chloroplast-encoded gene transcripts and photosynthetic protein accumulation**

**Fig. 6:**
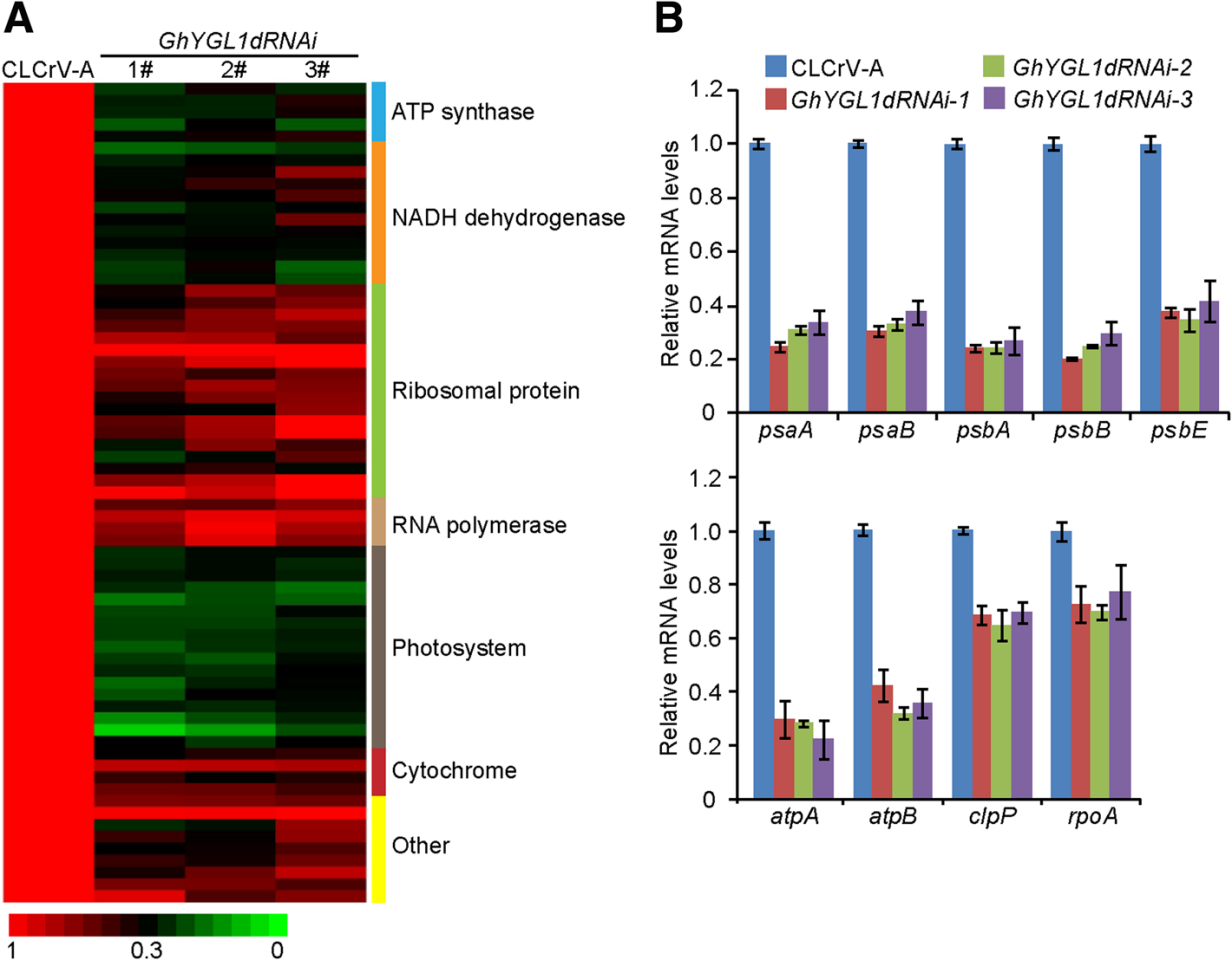
GhYGL1d regulates plastid function-related genes. **a** Plastid transcriptomic comparison of CLCrVA and GhYGL1d-RNAi variegated leaves. **b** Expression analysis of plastid-encoded genes in CLCrVA and GhYGL1d-RNAi plants. Transcription levels were measured via quantitative real-time RT-PCR, and *GhUBQ7* was used as a reference. Mean and SD values were obtained from three replicates.

**Fig. 7:**
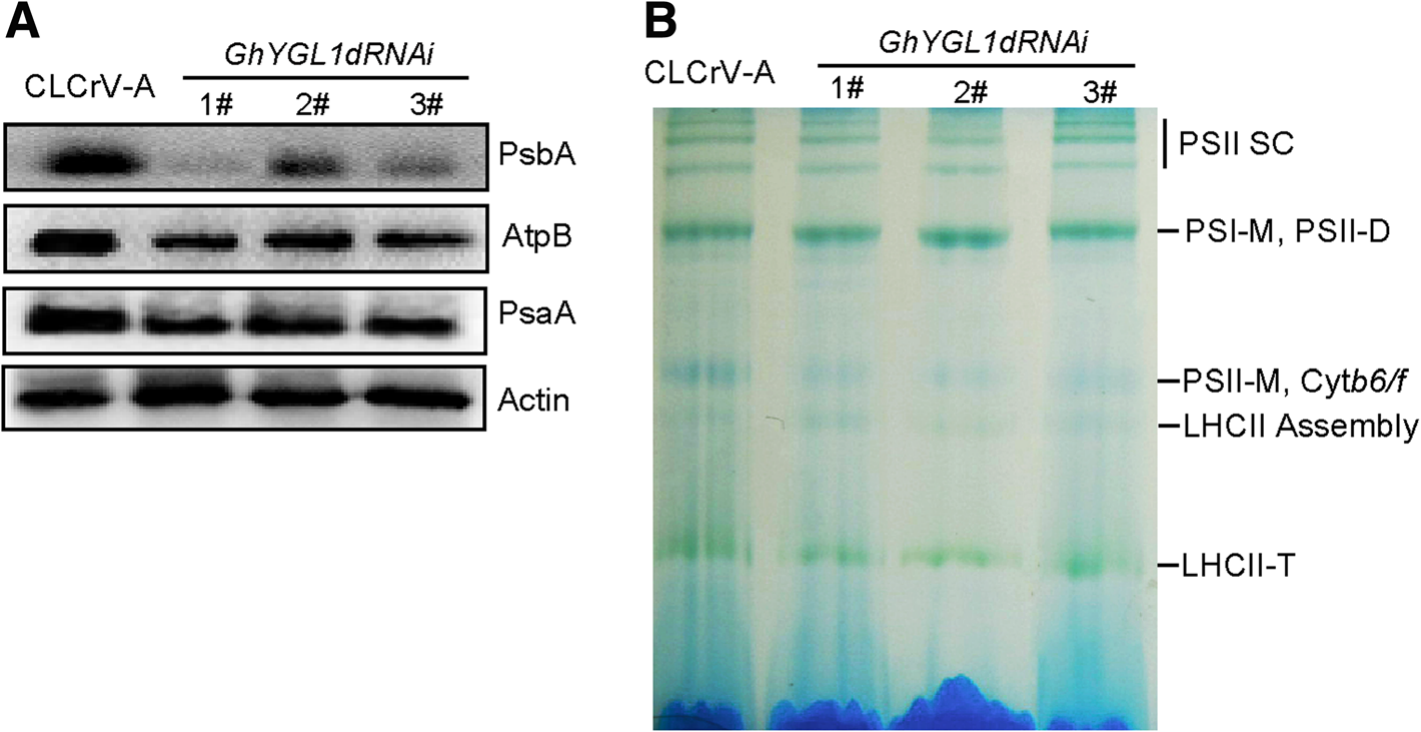
Analysis of photosynthetic complexes in RNAi plants and CLCrVA control plants. **a** Immunoblot analysis of photosynthetic proteins accumulated in CLCrVA and GhYGL1d-RNAi variegated leaves. Actin was used to check the difference in sample loading. **b** BN-PAGE analysis of photosynthetic complexes in CLCrVA and GhYGL1d-RNAi variegated leaves. Each lane was loaded with equal amounts of thylakoid membrane. PSI SC, PSI supercomplexes; PSI-M, PSI monomers; PSII-D, PSII dimers; PSII-M, PSII monomers; Cytb6/f, cytochrome f; and LHCII-T: PSII LHC trimers.

The expression of plastid-encoded genes is closely linked with chloroplast developmental status. To investigate whether defective chloroplast development was linked to the changes in gene expression, we performed RNA sequencing with samples prepared from control plants and three independent GhYGL1d RNAi lines. The genes expression of ATP synthase and NADH dehydrogenase and photosystems I and II were significantly reduced in GhYGL1d RNAi plants (Fig. 6a**,** Additional file 2 Table S3). In order to confirm the RNA-seq data, we examined transcription levels of some of these genes by RNA-qPCR. Photosystem I (PSI) subunit-*encoding* genes, psaA and psaB, and photosystem II (PSII) subunit-*encoding* genes, psaA, psaB and psaE, were drastically decreased in GhYGL1d RNAi plants compared with those in the WT. We noticed that the expression of ATP synthase genes (atpA and atpB) were significantly down-regulated in the RNAi plants, while the chloroplast caseinolytic protease gene ClpP and RNA polymerase subunit gene rpoA, exhibited a minimal reduction of expression in GhYGL1d RNAi plants (Fig. 6b). These results suggest that GhYGL1d may regulate the expression of plastid-encoded genes for chloroplast biogenesis. *To confirm whether the photosynthetic proteins are impaired in* GhYGL1d RNAi plants, we explored the *accumulation of photosynthetic proteins by immunoblot analyses. These results showed that* PSI, PSII and ATPase complex subunits *were clearly reduced in* GhYGL1d RNAi plants *(*Fig. 7a). Furthermore, we investigated the photosynthetic complexes of the thylakoid membrane in both *GhYGL1d RNAi* and WT *plants* by BN-PAGE. We found that the accumulation of PSII and PSI complexes was significantly reduced in *GhYGL1d RNAi plants compared with that in the WT* (Figure 7B).

## **Discussion**

Chloroplast development requires the balance between cytosolic and plastid co-expression and both nuclear- and chloroplast-encoding genes are coordinated to regulate chloroplast development [51]. In this study, we screened and identified a gene termed GhYGL1d, which regulates chloroplast development in cotton. GhYGL1d is a member of the PPR-DYW gene family and localized to the stroma fraction of chloroplasts. This gene might be involved in the C-to-U editing of accD and ndhF transcripts in cotton chloroplast. Down-regulation of GhYGL1d led to variegated leaves in cotton (Fig. 4a). These results suggest that GhYGL1d plays an important role in plastid development of cotton.

### **Loss of function of GhYGL1d causes defective chloroplast development**

The nucleus-encoded PPR proteins are important to plastid development. The loss-of-function of targeted plastid PPR proteins generally result in defects in chloroplast biogenesis. GhYGL1d is a typical PPR protein and *shared 11 conserved PPR motifs with AtECB2* of Arabidopsis. AtECB2 is required for early chloroplast biogenesis and the loss of its function resulted in the lack of thylakoid membranes, albino cotyledons and lethality of seedlings [52]. We demonstrated that *expressing* GhYGL1d *in the atecb2 mutant rescued cotyledons from albinism and the lethal* phenotype to be more similar to the wild type phenotype (Fig. 3a and b)*.* We also observed that the leaves of GhYGL1d-RNAi lines became a yellow-green variegated phenotype and had unorganized chloroplast structure. All these RNAi plants exhibited a significant reduction in *gene* transcription levels of PSI and PSII core subunits (psaA and psaB, psbA, psbB and psbE), and a concomitantly reduced protein expression levels of PSII supercomplexes. Photosystem II is a large protein complex in the membranes of chloroplasts, which is essential to plant growth. Some PSII proteins are encoded by nuclear genes and are regulated by post-transcriptional processes [53]. The psbA gene encoded the reaction center protein D1, which is important to the biogenesis and functional maintenance of PSII in higher plants. Multiple factors, PPR protein, LPE1, regulated D1 translation, and the down-expression of D1, resulted in a noticeable retardation of photoautotrophic growth [46]. Thus, the reduced growth and photosynthetic activity of GhYGL1d-RNAi plants probably resulted from comprehensive effects including inhibited assembly of PSI and PSII supercomplexes and other indirect targets. Additionally, we found that the transcription levels of both PEP dependent genes (PEPs) and NEP dependent genes (NEPs) were decreased in *GhYGL1*-silenced cotton (Fig. 6b), which is similar to the result that the transcription of PEPs (*psaA*, *psaB*, *psbA*, and *rbcL*) and NEPs ( *rpoA*, *rpoB*, *rps7* and *accD*) were reduced in rice al1 mutant [54]*.* There are two possible reasons, one is that the decreased transcription level of NEPs is the second effects of PEP reduction. The other is that chloroplast-to-nucleus retrograde signaling may affect the expression of nuclear encoded genes [55].

### **GhYGL1d may be involved in RNA editing of chloroplast genes**

Pentatricopeptide repeat proteins with a DYW domain in the C terminus were reported to be involved in RNA editing [47, 12]. For example, CRR22 and CRR28 genes played important roles in the editing of ndhB7, ndhD5 and rpoB3 [17]. In Arabidopsis, AtECB2 was involved in chloroplast transcript-RNA editing in accD and ndhF [50]. The gene accD encoded the b-subunit of the acetyl-CoA carboxylase complex, which is important for fatty acid synthesis. The gene ndhF encoded subunit F of NADH dehydrogenase. Both genes that lost their editing sites in transcripts corresponded to the albino phenotype in Arabidopsis [40]. In our research, RNA editing sites of accD and ndhF transcripts in the atecb2 mutant were rescued and more clearly resembled that of wild-type plants when the GhYGL1d gene was introduced into the mutant (Fig. 3d). This result suggests that GhYGL1d is involved in RNA editing of accD and ndhF transcripts. Both GhYGL1d and AtECB2 have similar sequences and might share the same biological functions in plastid development. We detected the editing events of plastid-genes transcripts and found that the editing efficiencies of accD-812 and ndhF-290 were reduced in CLCrVA-GhYGL1d RNAi plants compared with that in the *CLCrVA* plants (sss1 Fsigure S3). The results may be attributed to the cotton material used in the experiment: an RNAi plant in which the knock-down of GhYGL1d may have been insufficient to completely abolish RNA editing. As a result, the GhYGL1d RNAi cotton leaves exhibited yellow-green variegation instead of albinism. In the future, we plan to knock out GhYGL1d in cotton using CRISPR-Cas9 to further uncover its effects on RNA editing and assembly of PSI and PSII. In addition, determining which editing factors interact with GhYGL1d will help resolve the mechanisms of how GhYGL1d is involved in RNA editing.

## Conclusionss

The *GhYGL1d* gene has a DYW domain in C-terminal and is highly express in leaves, localized to the chloroplast fractions. Silencing of *GhYGL1d* led to the reduction of chlorophyll and phenotypically yellow-green leaves in cotton. Compared with wild type, *GhYGL1d*-silenced cotton showed significant deformations of thylakoid structures. Furthermore, the transcription levels of plastid-encoded polymerase- (PEP) dependent genes were significantly decreased in *GhYGL1d*-silenced cotton. Our data showed that *GhYGL1d* not only contributes to the editing of *accD* and *ndhF* genes, but also affects the expression of NEP- and PEP-dependent genes to regulate the development of thylakoids, and therefore regulates leaf variegation in cotton.

## Materials and Methods

### Plant Materials and Growth Conditions

*Gossypium hirsutum* (Xuzhou 142) seeds were planted into pots containing soil and grown in a climate chamber with 16:8 day:night light cycle at 30°C, as previously reported [56]. Two-week-old cotton plants were used for *Agrobacterium*-mediated VIGS assay. After inoculation, plants were transferred into a growth chamber set at 23°C. A total of nine seedlings were used for each treatment and three biological triplicates were performed per assay.

*Arabidopsis* T-DNA insertion lines of *atecb2* (SALK_112251, *AT1G15510*) in the Col-0 background were obtained from the ABRC (Arabidopsis Biological Resource Center) and identified by PCR with gene-specific primers. The plants were grown in standard soil in a growth chamber under a 12 h light/12 h dark cycle, 60–70% humidity, and a constant temperature of 22°C.

### Chlorophyll Content Measurement

The total chlorophyll content (ChI a+b) of leaves was measured as previous described [28]. Approximately 100 mg FW of leaf samples were ground in a mortar, then the homogenate was transferred to a tube with 5 ml of acetone 80% (v/v) and kept in the dark overnight. After that, the mixture were centrifuged at 2600 g for 15 min 4°C. The supernatant was transferred to a clean tube and used for chlorophyll content measurement. The chlorophyll content was determined by absorption measurements at 645 nm and 663 nm and calculated according to the following equation: C_a+b_ (mg/g) = [20.29*A*_645_+8.04*A*_663_] × *V*/1000 × *W*, where *V* represents the volume of the extraction buffer (ml) and *W* is the weight of the fresh leaves (g).

### Virus-induced gene silencing (VIGS)

To knock down the expression of *GhYGL1d*, a 325-bp fragment of *GhYGL1d* cDNA was PCR-amplified using TransStart FastPfu DNA Polymerase (Transgen, Beijing) and gene specific primers. The PCR product was cloned into the pCLCrVA vector to produce a VIGS vector named pCLCrVA-*GhYGL1d* and the empty pCLCrVA vector was used as control. The vectors pCLCrVB and pCLCrVA-*GhYGL1d* were introduced into the *Agrobacterium* strain LBA4404. For the VIGS array, the co-transformed *Agrobacterium* colonies containing pCLCrVB and pCLCrVA-*GhYGL1d* were grown for 24 h at 28°C on medium containing the proper antibiotic to select for transformants. *Agrobacterium* cells were collected and resuspended in infiltration medium (10 mM MgCl_2_, 10 mM MES, and 200 mM acetosyringone) and adjusted to an OD_600_ of 1.2. *Agrobacterium* cells containing pCLCrVB and pCLCrVA-*GhYGL1d* were mixed at a ratio of 1:1. The resuspended cells were injected into cotton cotyledons (approximately 10 days after germination) via a syringe. Thereafter, the plants were grown at 22°C in a growth chamber under a 16-h light, 8-h dark cycle.

### RNA isolation and quantitative RT-PCR analysis

Total RNA was isolated from cotton leaves using an RNA isolation kit (RNAqueous™ Total RNA Isolation Kit, Invitrogen™). RNA concentration was measured using a NanoDrop 2000 spectrophotometer (Thermo Scientific). The RNA was digested with RNase-Free DNase (Invitrogen) and checked for integrity by capillary gel electrophoresis. After that, total RNA was used to synthesize cDNA by One-Step gDNA Removal and cDNA Synthesis SuperMix (Transgen). Next, qRT-PCR was performed using SYBR Green PCR Master Mix (Takara). *GhUBQ7* was selected as the reference gene and each experiment had three biological repeats, each with three technical replicates.

### Subcellular localization of GhYGL1d

To investigate the subcellular localization of GhYGL1d, the open reading frame of *GhYGL1d* without a stop codon was then cloned into the pTF486 expression vector to generate a GhYGL1d-GFP fusion protein using a pEASY-Uni Seamless Cloning and Assembly Kit (Transgen). The fusion constructs GhYGL1d-GFP and empty vector 35S-GFP were introduced into *Arabidopsis* leaf protoplasts via polyethylene glycol-mediated transformation as described previously [57]. The transformed protoplasts were incubated at 23°C for 18 h. The fluorescence was visualized using a Leica TCS SP8 fluorescence confocal microscope. The GFP was visualized with excitation at 488 nm and emission at 505-530 nm. Chlorophyll fluorescence was visualized with excitation at 488 nm and emission at 650-710 nm, whereas the mitochondria was stained with MitoTracker^®^ Orange (M7510, Invitrogen) and visualized with excitation at 543 nm and emission at 560-600 nm.

### Chloroplast fractionation

Chloroplast fraction was isolated as described previously with modifications [36]. Briefly, 20-day-old plants were homogenized with ice cold extraction buffer (0.33 M sorbitol, 5 mM MgCl_2_, 5 mM EGTA, 5 mM EDTA, 50 mM HEPES-KOH pH 8.0 and 10 mM NaHCO_3_), the suspension was filtered through Miracloth and centrifuged at 2600 *g* at 4°C for 5 min. The pellets were resuspended in a extraction buffer and loaded onto Percoll step gradients (40%/70% Percoll, 0.33 M sorbitol, 2 mM EDTA, 50 mM HEPES, pH 8.0), and the collected chloroplast fractions were washed twice with wash buffer (0.33 M sorbitol and 50 mM HEPES-KOH, pH 8.0). Intact chloroplasts were further fractionated into stromal and thylakoid membrane fractions as previously reported [37].

### Transmission electron microscope (TEM) analysis

To assess the changes of plastid structure, we conducted a TEM analysis. The young leaves were fixed with 4% glutaraldehyde for over 48 h at 4°C and washed three times with PBS buffer. After that the sample was fixed with 1% osmic acid for 4 h and washed three times with PBS buffer. The sample was dehydrated using different concentration ethanol (30%, 50%, 70%, 90% and 100%). Next, it was embed and aggregated with ethoxyline resin and cut into slices. After stained with uranyl acetate and alkaline lead citrate for 15 min, the sample was observed using a Model H-7650 transmission electronmicroscope (HITACHI).

### Immunoblot analysis

Fresh leaves were ground into a fine powder in liquid nitrogen and used for protein extraction. The extracted protein samples were resolved on 12% SDS polyacrylamide gel electrophoresis (PAGE) gels, and then transferred to polyvinylidene difluoride membranes (0.45 μm, Millipore), followed by incubation with specific antibodies. The polyclonal antibodies of GFP, Cyt f, and RbcL were obtained from Agrisera.

### Thylakoid isolation and blue-native polyacrylamide gel electrophoresis

The Thylakoid was isolated as described previously with modification [38]. Thylakoids were isolated from fresh leaves with ice cold extraction buffer. The suspension was filtered through Miracloth and centrifuged at 2600 *g* at 4°C for 5 min. The pellet was resuspended in a wash buffer followed by centrifugation at 2400 *g* at 4°C for 5 min. Finally, the thylakoid pellet was suspended in storage buffer and treated with n-Dodecyl-beta-D-maltoside for 15 min. The insoluble material was removed by centrifugation at 16,000 *g* at 4°C for 10 min. For Blue Native-Polyacrylamide Gel Electrophoresis (BN-PAGE), 15 μg of solubilized thylakoid protein was mixed with a sample buffer and loaded onto a 4–15% native-PAGE gradient gel. Electrophoresis was performed at 4°C at a constant 120 V for 2.5 h.

## Additional file

Additional file 1: **Figure S1.** A virus-induced gene silencing (VIGS) assay for nine PPR-DYW genes. **Figure S2.** Alignment of amino acid sequences of the highest identity with the GhYGL1d protein. **Figure S3.** The editing efficiency of *accD*-812 and *ndhF*-290 in CLCrVA and GhYGL1d-RNAi plants. **Table S1.** Subcellular localization prediction of 72 PPR-DYW proteins in cotton. **Table S2.** Primer sequences used in this study (PDF 1207 kb)

Additional file 2: **Table S3.** RNA-seq dataset of CLCrVA and GhYGL1d-RNAi plants. (XLSX 17526 kb)

## Data Availability

The sequence data during the current study could be found in COTTONGEN (https://www.cottongen.org), The transcriptome data supporting the results of this article is contained within supplementary information files (Table S3).

## Abbreviations

PPR: pentatricopeptide repeat
VIGS: virus-induced gene silencing
GFP: green fluorescent protein
TEM: transmission electron microscope
PEP: plastid-encoded polymerase
NEP: nuclear-encoded polymerase
BN-PAGE: blue native polyacrylamide gel electrophoresis
cTP: chloroplast transit peptide
