# Supplementary material for: GhYGL1d, a pentatricopeptide repeat protein, is required for chloroplast development in cotton
Source: BMC Plant Biol. 2019 Aug 13;19:350. doi: 10.1186/s12870-019-1945-1 (PMC6693126; doi:10.1186/s12870-019-1945-1)
Supplement: Supplementary file 1 — Figure S1. A virus-induced gene silencing (VIGS) assay for nine PPR-DYW genes. Figure S2. Alignment of amino acid sequences of the highest identity with the GhYGL1d protein. Figure S3. The editing efficiency of accD-812 and ndhF-290 in CLCrVA and GhYGL1d-RNAi plants. Table S1. Subcellular localization prediction of 72 PPR-DYW proteins in cotton. Table S2. Primer sequences used in this study (PDF 1207 kb) [file 12870_2019_1945_MOESM1_ESM.pdf]

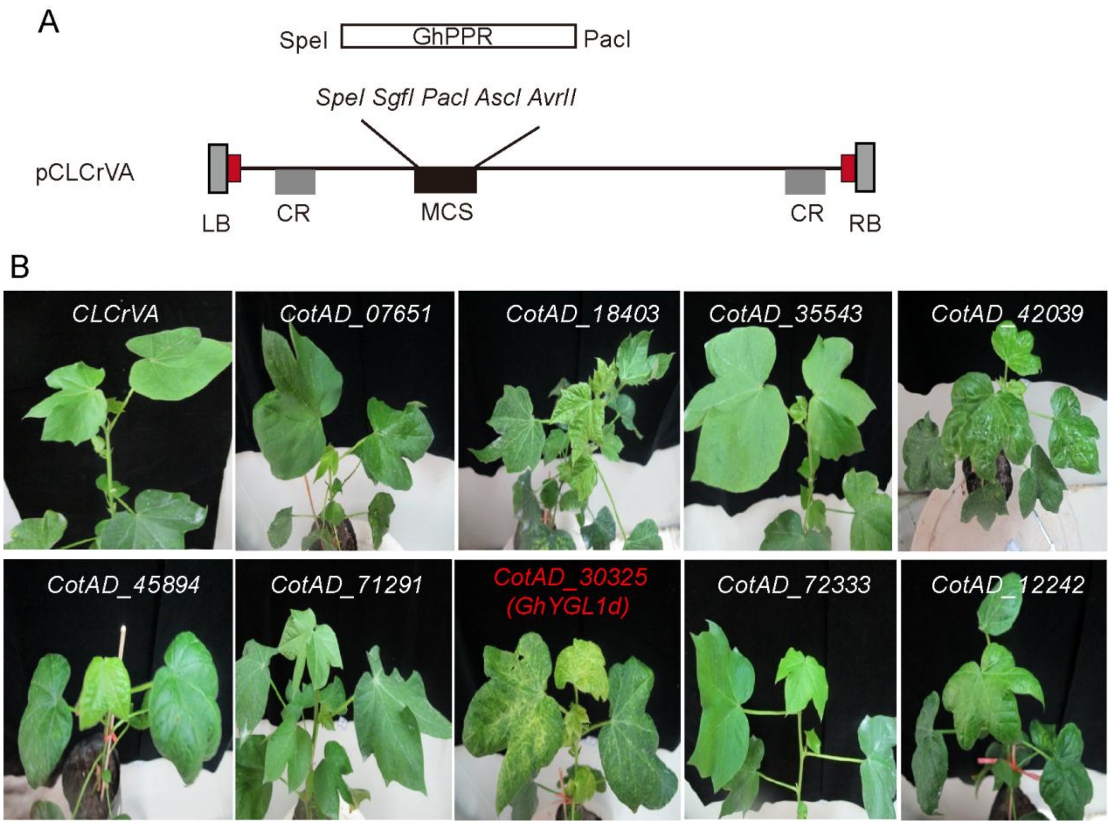

Figure S1 A virus induced gene silencing (VIGS) assay for 9 PPR-DYW genes.

A. Schematic illustration of the pCLCrVA-PPR construts.

B. The phenotypes in the leaves of nine PPR-DYW genes silencing cotton plants. CLCrVA (empty vector) was used as a negative control.

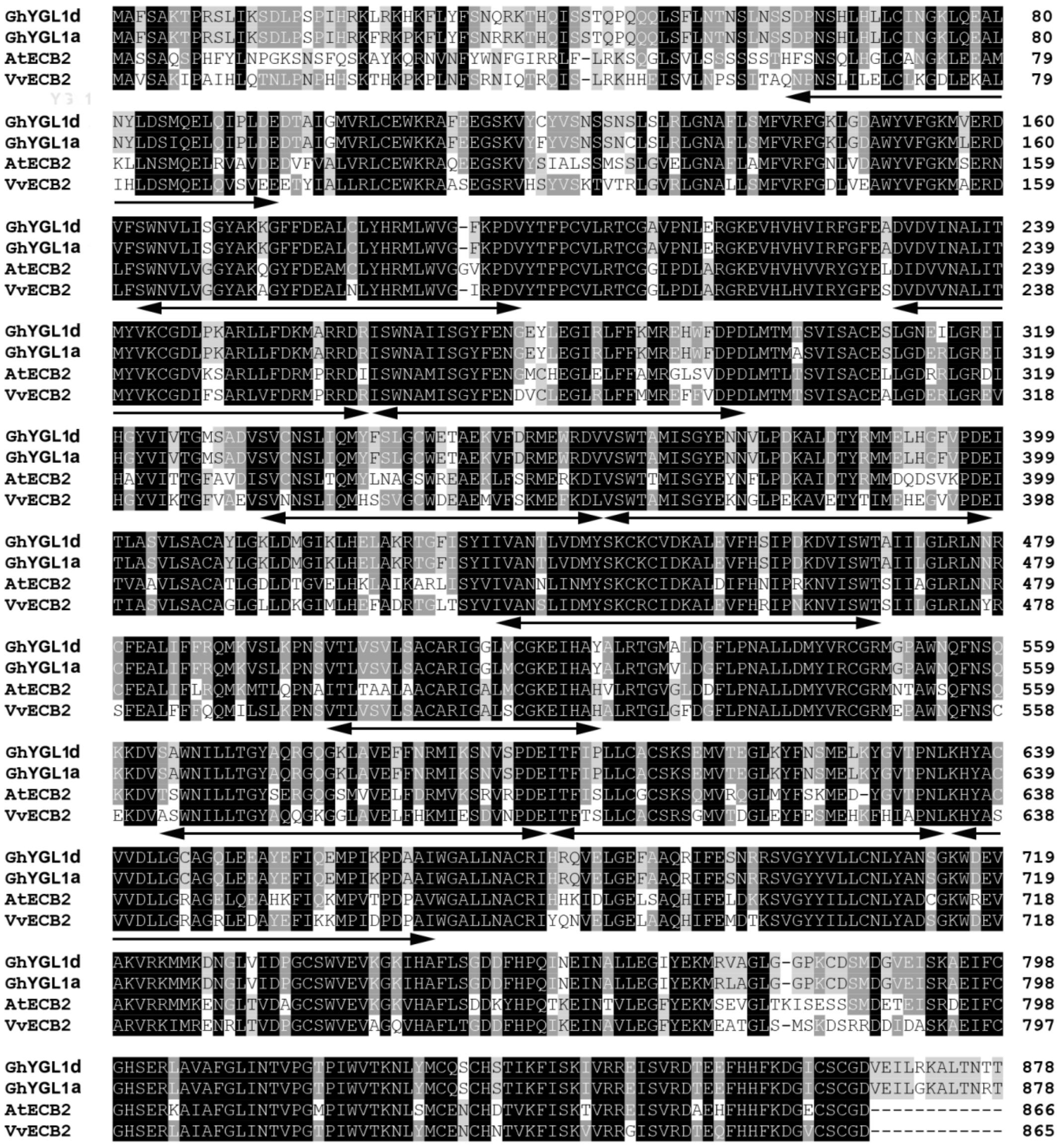

Figure S2 Alignment of amino acid sequences with the highest identity with the GhYGL1 protein.

The *Arabidopsis* homolog is annotated as AtECB2. GenBank accession numbers for the other protein sequences are: GhYGL1d (CotA_30325); GhYGL1a (CotA_60660); AtECB2 (NC_003070.9); VvECB2 (XP_002285225.2). Conserved amino acids are highlighted in shades of black and the two deleted amino acids indicated by black boxes are highly conserved in these species. PPR repeats are indicated below the sequences by double-headed arrows.

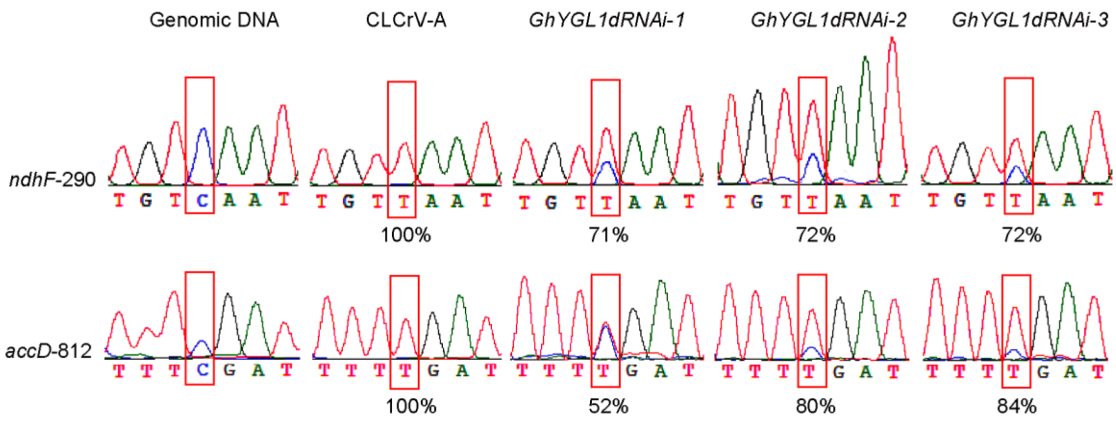

Figure S3 The editing efficiency of *accD*-812 and *ndhF*-290 in CLCrVA and GhYGL1d-RNAi plants. The editing efficiency of C-to-U was calculated by the peak value of T (hT) and T + C (hC+ hT) at the same site, namely editing frequency = hT/(hC + hT) ×100%.

Table S1. Subcellular localization prediction of 72 PPR-DYW proteins in cotton.

Gene number	Amino acids	PPR motifs	Location	cTP/mTP/SP value
CotAD_35543	1147	9	C	0.868
CotAD_60660	882	11	C	0.858
CotAD_18403	883	7	C	0.858
CotAD_07651	1066	12	C	0.792
CotAD_42039	721	8	C	0.758
CotAD_30325	882	12	C	0.705
CotAD_45894	803	9	C	0.676
CotAD_72333	891	9	C	0.676
CotAD_71291	840	11	C	0.674
CotAD_12242	773	8	C	0.62
CotAD_60388	611	6	C	0.559
CotAD_30767	749	7	C	0.551
CotAD_52513	826	12	C	0.522
CotAD_64538	623	9	C	0.518
CotAD_23452	788	8	C	0.504
CotAD_14466	644	6	C	0.494
CotAD_34336	702	9	C	0.478
CotAD_36806	659	9	C	0.281
CotAD_30125	892	12	C	0.269
CotAD_10910	1078	10	C	0.246
CotAD_24153	823	10	M	0.907
CotAD_24956	376	4	M	0.819
CotAD_02921	656	7	M	0.788
CotAD_45531	680	4	M	0.73
CotAD_23421	630	7	M	0.637
CotAD_04083	750	10	M	0.57
CotAD_15566	868	11	M	0.533
CotAD_62766	868	11	M	0.526
CotAD_63586	866	11	M	0.526
CotAD_06720	612	8	M	0.465
CotAD_42662	774	9	M	0.391
CotAD_29636	940	13	M	0.369
CotAD_65621	937	13	M	0.288
CotAD_42777	1741	22	M	0.288
CotAD_12486	754	8	M	0.176
CotAD_48709	485	7	S	0.971
CotAD_38628	799	13	S	0.909
CotAD_10473	218	1	S	0.908
CotAD_04368	611	6	S	0.846
CotAD_05515	243	2	S	0.815

CotAD_18473	754	10	S	0.751
CotAD_40832	918	10	S	0.689
CotAD_72146	786	9	S	0.655
CotAD_12790	452	5	S	0.643
CotAD_63545	644	9	S	0.442
CotAD_00143	610	5	S	0.223
CotAD_12322	1243	13	*	-
CotAD_34740	748	12	*	-
CotAD_16490	855	11	*	-
CotAD_34536	813	10	*	-
CotAD_63199	735	10	*	-
CotAD_28387	643	8	*	-
CotAD_44816	685	7	*	-
CotAD_55201	755	8	*	-
CotAD_02159	692	10	*	-
CotAD_57064	583	5	*	-
CotAD_18938	622	8	*	-
CotAD_20522	586	7	*	-
CotAD_61963	696	10	*	-
CotAD_22605	645	8	*	-
CotAD_20539	579	6	*	-
CotAD_69858	688	7	*	-
CotAD_05547	430	7	*	-
CotAD_02332	489	9	*	-
CotAD_04074	441	6	*	-
CotAD_14507	403	6	*	-
CotAD_75610	332	3	*	-
CotAD_27114	334	5	*	-
CotAD_43221	490	4	*	-
CotAD_41541	246	2	*	-
CotAD_37496	609	8	*	-
CotAD_47098	1165	11	*	-

C, Chloroplast; M, Mitochondria; "*", unknown localization.

SP, a signal peptide. mTP, a mitochondrial targeting peptide; cTP, a chloroplast targeting peptide; "-", Any other location.

Table S2. Primer sequences used in this paper.

Primer name	Sequence（5'→3'）
CotAD_71291-clv-F	GGACTAGTATGGAGGGTTAGTCGAGCAA
CotAD_71291-clv-R	CGTTAATTAAACCTCTATTGAGCTTGATCCAG
CotAD_72333-clv-F	GGACTAGTAACCATGGCCAGAGCACTAT
CotAD_72333-clv-R	CGTTAATTAATTCCCCACCTCAATCCAACT
CotAD_35543-clv-F	GGACTAGTCGATATCCACTCTCGATGTCA
CotAD_35543-clv-R	CGTTAATTAAGAAGCAATACTCATTTGGCA
CotAD_45894-clv-F	GGACTAGTTCAAACATCTTAGCCGAACC
CotAD_45894-clv-R	CGTTAATTAATCTCTCCCAATTCAACGCAC
CotAD_18403-clv-F	GGACTAGTATCAGGGAGATGGCATTGCT
CotAD_18403-clv-R	CGTTAATTAAACCAGAAGCATCCCTCACTA
CotAD_07651-clv-F	GGACTAGTTCATGTAACCTTCGTCGGTG
CotAD_07651-clv-R	CGTTAATTAATCTGCTACACCATCCCAGTT
CotAD_30325-clv-F	GGACTAGTTATGTTTGTGAGGTTTGGGA
CotAD_30325-clv-R	CGTTAATTAACTTATCCCTTCCAAATACTC
CotAD_12242-clv-F	TCTACTAGTCAGTGGCAAATAGGTGGAATG
CotAD_12242-clv-R	TCATTAATTAATTCCGTCTCTGAAGTGGTGA
CotAD_42039-clv-F	GGACTAGTTATGCTTGTAGCCATGCAGG
CotAD_42039-clv-R	CGTTAATTAATTGCCTCAAGTCTCCAACAT
GhYGL1d-F	GTTAGGGATACTGAAGAATTCCACCA
GhYGL1d-R	TAACTGGCTGATCCATGATGCATA
GhUBQ7-SP1	GGCATTCCACCTGACCAACAA
GhUBQ7-AP1	CCGCATTAGGGCACTCTTTTC
